# Magnetoresistance Anomaly in Topological Kondo Insulator SmB_6_ Nanowires with Strong Surface Magnetism

**DOI:** 10.1002/advs.201700753

**Published:** 2018-05-21

**Authors:** Xingshuai He, Haibo Gan, Zongzheng Du, Bicong Ye, Liang Zhou, Yuan Tian, Shaozhi Deng, Guoping Guo, Haizhou Lu, Fei Liu, Hongtao He

**Affiliations:** ^1^ Institute for Quantum Science and Engineering and Department of Physics South University of Science and Technology of China Shenzhen 518055 China; ^2^ State Key Laboratory of Optoelectronic Materials and Technologies Guangdong Province Key Laboratory of Display Material and Technology and School of Electronics and Information Technology Sun Yat‐sen University Guangzhou 510275 China; ^3^ School of Physics Southeast University Nanjing 211189 China; ^4^ Key Laboratory of Quantum Information CAS University of Science and Technology of China Hefei 230026 China

**Keywords:** Kondo breakdown, magnetic ordering, magnetotransport, topological Kondo insulators, topological surface states

## Abstract

Topological Kondo insulators (TKIs) are a new class of topological materials in which topological surface states dominate the transport properties at low temperatures. They are also an ideal platform for studying the interplay between strong electron correlations and topological order. Here, hysteretic magnetoresistance (MR) is observed in TKI SmB_6_ thin nanowires at temperatures up to 8 K, revealing the strong magnetism at the surface of SmB_6_. It is also found that such MR anomaly exhibits an intriguing finite size effect and only appears in nanowires with diameter smaller than 58 nm. These nontrivial phenomena are discussed in terms of the latest Kondo breakdown model, which incorporates the RKKY magnetic interaction mediated by surface states with the strong electron correlation in SmB_6_. It would provide new insight into the nature of TKI surface states. Additionally, a non‐monotonically temperature dependent positive magnetoresistance is observed at intermediate temperatures, suggesting the possible impurity‐band conduction in SmB_6_, other than the surface state transport at low temperatures and the bulk‐band transport at high temperatures.

## 1 Introduction

As a new quantum state of matter, topological insulators (TIs) host spin‐helical surface states protected by time reversal symmetry.^1^, ^2^ Many intriguing phenomena such as weak anti‐localization, quantum spin, and anomalous Hall effect have been discovered in TIs.^3^, ^4^, ^5^, ^6^ The interplay between TIs and magnetism or superconductivity can even lead to the possible realization of magnetic monopoles^7^ or Majorana fermions.^8^ Therefore, the study of topological insulators has been one of the recent focuses of condensed matter physics and material science. But the dominant bulk conduction in most TIs hinders the direct investigation of theses novel surface states. This makes topological Kondo insulators (TKIs), a new class of TIs, especially important since the transport of TKIs is dominated by topological surface states at low temperatures.

TKIs are topologically ordered and strongly correlated materials with dense arrays of screened local moments. SmB_6_ is a typical candidate of such TKIs. SmB_6_ has long been known as Kondo insulators since the Kondo screening of Sm localized f‐electrons by mobile d‐electrons can lead to an insulating bulk hybridization gap in SmB_6_, which is manifested by a rapid increase of resistivity with decreasing temperatures.^9^ Recently, it is recognized that this hybridization of odd‐parity f‐electrons with even‐parity d‐electrons can result in the TKI phase in SmB_6_ at low temperatures where the hybridization gap fully opens.^10^ The existence of metallic surface states has been verified in various transport studies of SmB_6,_ such as the capacitive self‐oscillation experiment,^11^ the point‐contact spectroscopy,^12^ the nonlocal and thickness independent transport measurement.^13^, ^14^ ARPES measurement also reveals the three surface Dirac cones located at the Γ and two X points, respectively.^15^ The linear energy dispersion and spin texture of surface states were also studied in SmB_6_.^16^, ^17^, ^18^

Although much progress has been made in the study of TKI phase in SmB_6_, some puzzling phenomena remain to be resolved. A recent quantum oscillation experiment reveals a 3D bulk Fermi surface in TKI SmB_6_, instead of 2D one as expected.^19^, ^20^ Surface magnetic ordering is also detected in the latest hysteretic magnetoresistance (MR) measurement of SmB_6_ crystals below 600 mK.^21^ It might lead to possible chiral edge state transport in SmB_6_, but the physical origin of it is still under investigation. A latest Kondo breakdown scenario has been proposed to account for the above experiments.^22^, ^23^ Although such a scenario would provide new insights into the nature of the TKI phase in SmB_6_, the validity of it needs further experimental examination. In this work, we have performed systematic magneto‐transport study of thin SmB_6_ nanowires (NWs) with diameters down to 45 nm. With the hybridization gap fully opening, an MR anomaly indicating the emergence of surface magnetism is clearly observed in our work, whose characteristic temperatures (8 K) is at least one order of magnitude higher than previous study (600 mK).^21^ This MR anomaly is also found to show a nontrivial dependence on the diameter of NWs. The possible role of surface Kondo breakdown in this MR anomaly is discussed, revealing the important interplay between magnetic interaction and Kondo screening at the surface of TKI SmB_6_. Furthermore, a low‐field positive MR is also observed at intermediate temperatures, indicative of impurity band conduction in SmB_6_, other than the bulk and surface state transport. All these results demonstrate the complexity and novelty of surface states in TKI SmB_6_ and are essential to clarify the subtle nature of the TKI phase in SmB_6_.

## 2 Results and Discussion

### 2.1 Synthesis and Characterization of SmB_6_ Nanowires

**Figure 1:**
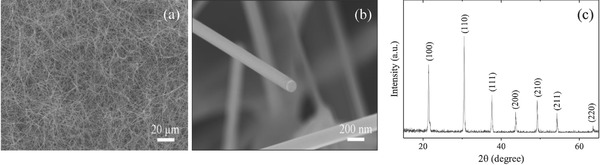
a,b) SEM images of SmB_6_ NWs obtained at low and high magnifications. c) XRD pattern of SmB_6_ NWs.

**Figure 2:**
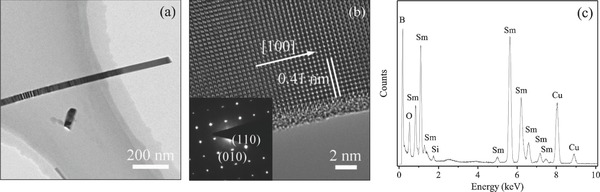
TEM images of a SmB_6_ NW: a) TEM image, b) HRTEM image, and c) EDX spectrum. Inset of (b) shows the SAED pattern.

The SmB_6_ NWs were grown by simple thermal‐reduction way (see the Experimental Section for details).^24^, ^25^, ^26^ **Figure** **1**a shows the scanning electron microscopy (SEM) image of the obtained SmB_6_ NWs. High‐magnification SEM image of a typical SmB_6_ NW is given in Figure 1b, clearly demonstrating the round end of the NW. The NW diameter (*D*) ranges from 40 to 1540 nm. Moreover, sharp and well‐defined diffraction peaks are found in the X‐ray diffraction (XRD) pattern obtained from these NWs in Figure 1c, which agrees with the SmB_6_ cubic crystal structure with the space group of $P\bar{m}3m$. The transmission electron microscopy (TEM) measurements also reveal the high crystallinity of our SmB_6_ NWs. **Figure** **2**a is the low‐magnification TEM image of a SmB_6_ NW with *D* of 40 nm. High‐resolution TEM (HRTEM) image along the edge of the NW clearly shows the cubic symmetry of the crystal structure, as seen in Figure 2b. The growth direction of the NW is thus determined to be the [100] direction with the *d*‐spacing value of 0.41 nm, as indicated in Figure 2b. These results are consistent with the selected area electron diffraction (SAED) pattern shown in the inset of Figure 2b, where sharp and clear spots can be found. It is also noticed in Figure 2b that an amorphous layer with a thickness about 1 nm appears at the surface of the SmB_6_ NW. Based on the energy dispersive X‐ray spectroscopy (EDX) spectrum (Figure 2c), very small amount of O element was observed in the SmB_6_ NW, revealing that this amorphous layer arises from the surface oxidization of the SmB_6_ nanowire.^27^ Based on the HRTEM and EDX results, the as‐grown nanowires can be proven to be SmB_6_ single crystals with a growth direction of [100].

### 2.2 Magnetoresistance Anomaly below 8 K

**Figure 3:**
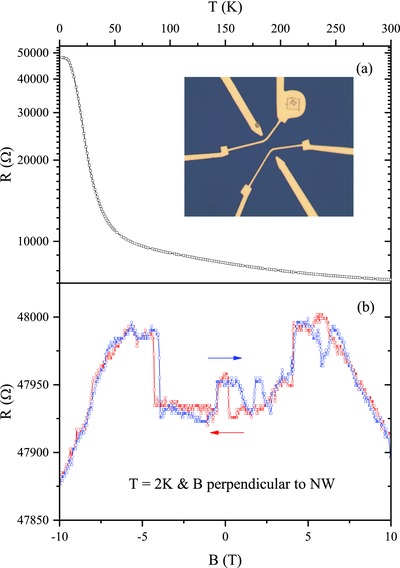
a) The temperature dependence of resistance of a SmB_6_ nanowire with *D* of 45 nm. The inset shows the image of a fabricated four‐probe device. b) Double‐sweep magnetoresistance curve with *B* perpendicular to NW and *T* = 2 K. The field sweeping directions are indicated by arrows.

**Figure** **3**a shows the temperature (*T*) dependence of resistance (*R*) of a SmB_6_ nanowire with *D* of 45 nm. With temperatures decreasing below 50 K, a rapid increase of resistance is observed, indicating the gradual opening of a bulk energy band gap due to the hybridization between Sm 4f and 3d electrons.^9^ Further decreasing the temperature leads to the saturation of the *R*(*T*) curve below 10 K. Such a resistance saturation was a long puzzling question in the study of Kondo insulator SmB_6_ and previously ascribed to the impurity band conduction.^28^ But recently it is regarded as a transport signature of topological surface states in the TKI phase of SmB_6_ when the hybridization gap fully opens.^10^

Figure 3b shows the double‐sweep MR curves in perpendicular magnetic field (*B*) and at *T* = 2 K, with the field sweeping directions indicated by red and blue arrows. Surprisingly, the obtained *R*(*B*) curves depend on the sweeping directions and exhibit hysteretic behavior. Besides this, sharp changes in MR are also clearly observed in the *R*(*B*) curves, e.g., at about ±4T. Such abnormal features are in sharp contrast with the smooth and nonhysteretic MR obtained in high magnetic field region (*B* > 8T), as shown in Figure 3b. The high‐field negative MR is generally understood as the suppression of the hybridization gap by magnetic fields.^29^ But this mechanism is apparently not applicable to the MR anomaly observed in the low‐field region.

**Figure 4:**
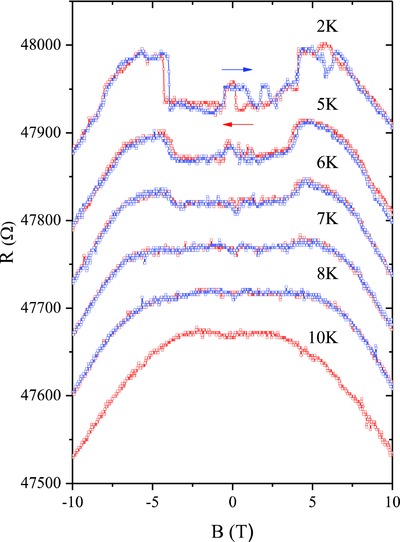
Double‐sweep MR curves of a SmB_6_ NW in perpendicular magnetic fields at different temperatures. The NW diameter is 45 nm and the field sweeping directions are indicated by arrows. Curves are offset vertically for comparison. At *T* = 10 K, the MR curve was only measured in the sweep‐down direction.

In order to study the possible physical origin underlying this low‐field MR anomaly, we also measured the MR of the SmB_6_ nanowire at different low temperatures, as shown in **Figure** **4**. For clarity, double‐sweep MR curves obtained at different temperatures are offset vertically. With temperatures increasing from 2 to 8 K, the magnitude of the sharp change in MR decreases gradually and the hysteretic behavior disappears. At *T* = 10 K, the measured MR curve is rather smooth and shows no discernible hint of the MR anomaly, except a weak positive MR in zero magnetic fields, which will be discussed later. Therefore, the MR anomaly persists up to at least 8 K, where the hybridization gap fully opens and the surface state dominates the transport properties of SmB_6_. It is also worth pointing out that in comparison with the low‐field MR anomaly, the high‐field negative MR shows little changes with increasing temperatures, indicating the different physical origins of the low‐field and high‐field MR.

**Figure 5:**
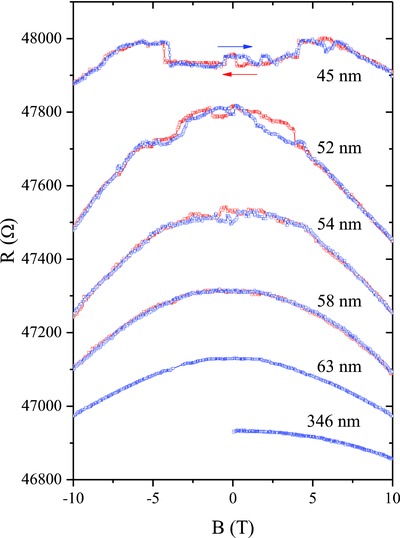
Double‐sweep MR curves of different SmB_6_ NWs in perpendicular magnetic fields at *T* = 2 K. The NW diameters and field sweeping directions are as indicated. All curves are offset vertically for clarity. The MR curves of NWs with *D* = 63 and 346 nm were only measured in the sweep‐up direction.

Furthermore, the low‐field MR anomaly shows a striking dependence on the NW diameter. **Figure** **5** shows the double‐sweep MR curves of NWs with different diameters at *T* = 2 K. The curves have been offset for clarity and the field sweeping directions are indicated by arrows. As shown in the figure, similar MR anomaly is clearly observed in low fields in another two thin NWs with *D* of 52 and 54 nm. But it gradually weakens with increasing diameter of the NWs and totally disappears in thick NWs with *D* of 58, 63, and 346 nm. This suggests the finite size effect on the low‐field MR anomaly.

The appearance of the MR anomaly in low field region and its systematic dependence on *T* and *D* clearly demonstrate that the observed MR anomaly is a real physical phenomenon occurring in thin SmB_6_ NWs when surface states dominate the transport properties of SmB_6_. We have also measured the SmB_6_ nanowires with cycles of cooling down and warming up. The MR anomaly still persists at low temperatures under such temperature cycling (Figure S1, Supporting Information). This MR anomaly strongly indicates the emergence of surface magnetism in TKI SmB_6_. It is noted that similar results have been reported in latest transport studies of SmB_6_ bulk crystals and surface magnetism has been ascribed to surface oxide layer or Kondo holes.^21^, ^30^ According to the HRTEM image in Figure 2b, there is apparently an amorphous oxide layer at the surface of our crystalline SmB_6_ NWs. It is thus appealing to attribute the surface magnetism in our SmB_6_ NWs to the surface magnetic oxide layer. But our work does show differences with previous studies. First, the phenomena of previous works only appear at very low temperatures (*T* < 600 mK), which is at least one order of magnitude smaller than ours, where the MR anomaly persists up to 8 K (see Figure 4). Second, this high‐temperature MR anomaly exhibits a finite size effect as shown in Figure 5. It can only be observed in thin NWs, but not in thick ones, although all NWs in our work have surface oxide layers. These discrepancies make us believe that there must be some other physical mechanisms responsible for the surface magnetism in our thin SmB_6_ NWs.

Besides the extrinsic surface magnetism,^21^, ^30^ latest theoretical studies have revealed that Kondo breakdown at the surface of SmB_6_ can be a possible intrinsic origin of surface magnetism in TKI.^22^, ^23^ The Kondo breakdown was originally proposed to understand the puzzle that a 3D bulk Fermi surface was detected in a quantum oscillation experiment of SmB_6_ at low temperatures where the bulk hybridization gap fully opens.^20^ Due to reduced coordination number of Sm ions at the surface, it is predicted that the surface Sm ions will be less screened in comparison with the bulk ones during the opening of the hybridization gap. At temperatures where the resistivity of SmB_6_ saturates, the bulk hybridization gap fully opens and topological surface states dominate the transport properties of SmB_6_. The RKKY interaction between the surface Sm local moments and the topological surface states will dominate over the Kondo screening at the surface of SmB_6_. In such a situation, the surface of SmB_6_ can be described by the chiral Anderson model, the ground state of which could be magnetic.^22^ Such an intrinsic scenario for surface magnetism is in agreement with our results shown in Figure 4, i.e., the MR anomaly begins to appear with the fully opening of the hybridization gap below 10 K. Therefore, the MR anomaly in our work is observed at temperatures much higher than those in previous studies where surface magnetism was ascribed to extrinsic origins.^21^, ^30^

From the above discussion, it can also be seen that the RKKY interaction competes with the Kondo screening at the surface of SmB_6_. The interplay between RKKY interaction and Kondo screening at the surface determines the physics of the TKI phase in SmB_6_.^31^, ^32^ As shown in Figure 5, the low‐field MR anomaly indicating the emergence of surface magnetism only appears in thin SmB_6_ NWs, but vanishes in thick ones. This is in sharp contrast with conventional FM materials, where reduced dimension tends to suppress long‐range magnetic order based on the Mermin–Wagner theorem.^33^ Such a nontrivial dependence on the diameter of SmB_6_ NWs might be another consequence of the Kondo breakdown in SmB_6_. As pointed out in previous studies, Kondo effect exhibits a finite size effect and is greatly suppressed in low dimensional systems.^34^, ^35^ Therefore, for the surface Sm ions, not only reduced coordination number, but also reduced size can lead to the suppression of Kondo screening, i.e., surface Kondo screening in SmB_6_ is expected to decrease with decreasing *D*. This will result in weaker Kondo screening (or enhanced Kondo breakdown) and stronger RKKY interactions at the surface of thin SmB_6_ NWs. The MR anomaly is thus more likely to be observed in thin NWs. It is unlikely that the nontrivial diameter dependence of the MR anomaly arises from the diameter‐dependent doping. The doping would certainly influence the bulk properties of SmB_6_, but at low temperatures, the bulk is insulating and the transport is only dominated by the surface states. The Fermi levels of all the samples we studied stay in the hybridization gap below 10 K. This gap is only about 10–20 meV,^36^ i.e., the difference between the Fermi energy of our samples should be smaller than 10–20 meV. The doping is thus expected to play a minor role in the diameter dependent MR anomaly. Future electrostatic gating experiment would help to clarify this issue.^36^

At present stage, it is believed that the intrinsic Kondo breakdown is crucial to the observation of the MR anomaly up to 8K in SmB_6_ NWs. Our magnetotransport results clearly demonstrate that the surface states of SmB_6_ are far more complex than those of topological insulators, such as Bi_2_Se_3_ and Bi_2_Te_3_. The nontrivial NW diameter dependence shown in Figure 5 also reveals that the system dimension plays an important role in the Kondo breakdown model. Since the surface disorder will greatly influence the surface magnetic state in both extrinsic and intrinsic scenarios,^22^, ^23^, ^30^ this will further complicate the analysis of this intriguing phenomenon in SmB_6_. The interplay between magnetic interaction, Kondo screening, and disorder at the surface of SmB_6_ is thus essential to the fully understanding of the physics in TKIs. We also note that although the Kondo breakdown model captures the main topological properties of SmB_6_, it is not a sufficient minimal model of SmB_6_, which contains three 4f orbitals and two 5d orbitals near the Fermi level.^37^ The 4f orbitals of Sm are split into several multiplets. Considering the symmetry of the multiplets, only two 4f orbitals are allowed to hybridize with the 5d orbitals via the Kondo effect.^37^, ^38^ In principle, it is essential to include more orbitals to quantitatively describe SmB_6_ and the emergent surface magnetism. Thus, further theoretical studies are needed to gain a deeper insight and quantitative physical picture of the emergent surface magnetism in SmB_6_.

### 2.3 Positive Magnetoresistance at Intermediate Temperatures

**Figure 6:**
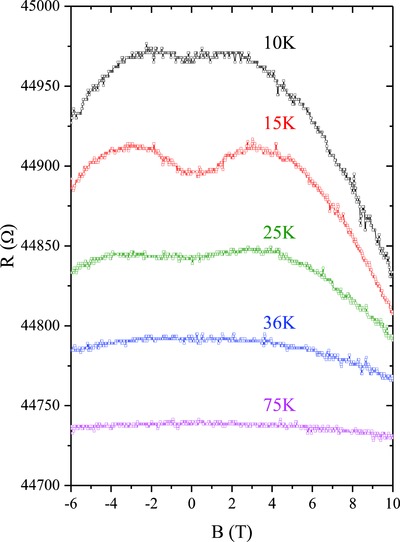
MR curves of SmB_6_ NWs in perpendicular magnetic fields at different temperatures. The NW diameter is 45 nm. All curves are offset vertically for clarity.

Besides the MR anomaly, the SmB_6_ NW is also found to exhibit a low‐field positive MR when the temperature is increased above 10 K. As shown in **Figure** **6**, a positive MR begins to appear at *T* = 10 K and reaches the maximum at *T* = 15 K. Further increasing the temperature weakens the positive MR and it disappears at *T* = 36 K. No such positive MR is observed at higher temperatures, as shown by the MR curve obtained at *T* = 75 K. Therefore, the positive MR displays a non‐monotonic dependence on *T* and only appears in the temperature range where the bulk hybridization gap develops. Usually negative MR is anticipated for the bulk‐state transport in SmB_6_ since the applied field can suppress the bulk hybridization gap.^29^ The non‐monotonic temperature dependence also excludes the weak antilocalization and linear magnetoresistance arising from the surface state in SmB_6_.^39^ After ruling out possible contributions from the bulk and surface states of SmB_6_, we tentatively ascribe this phenomenon to the impurity‐band conduction in SmB_6_.^28^ At intermediate temperatures when the hybridization gap gradually forms, carrier transport may occur via the variable range hopping in the impurity band. A positive MR is expected since the localization length can be reduced by the magnetic field.^40^ Similar impurity band related positive MR has been discussed in the study of Ce_1‐_ *_x_*La*_x_*Os_4_Sb_12_ alloys, a possible Kondo insulator.^41^ At higher or lower temperatures, bulk or surface state transport will dominate over the impurity band conduction. This explains the non‐monotonic temperature dependence of the positive MR shown in Figure 6.

## 3 Conclusion

In conclusion, a low‐field MR anomaly is observed in SmB_6_ NWs in the resistivity saturation region where the hybridization gap fully opens. It persists up to 8 K and shows a nontrivial dependence on the diameter of NWs. Such phenomena might be a manifestation of the proposed Kondo breakdown picture, which is important to the interpretation of surface state properties in TKI SmB_6_. At intermediate temperatures, the appearance of low‐field positive MR indicates the impurity band conduction in SmB_6_, other than the low‐temperature surface state transport and high‐temperature bulk state transport. Our results clearly demonstrate that the physics of TKI SmB_6_ is not only governed by Kondo screening, but also by magnetic interaction as well as disorder.

## 4 Experimental Section

*Growth*: The SmB_6_ NWs were grown by simple thermal‐reduction way. In the fabrication process, SmCl_3_, boron, and boron oxide powders were used as the source materials. Ni film was deposited on the surface of Si substrate to be the catalyst of the formation of the SmB_6_ NWs. The mixed gas of Ar and H_2_ was chosen as the carrier gas in the reaction, in which the chamber pressure was kept at 0.25–0.75 atmosphere. This reaction lasted for 1–2 h at 1000–1100 °C. After the growth was over, a dark‐blue film was found on the surface of Si substrate.

*Characterization*: SEM (Zeiss, SUPER‐55) and TEM (FEI, Titan3 G2 60–300) were respectively used to investigate the morphology and crystalline structure of the sample. And XRD (Rigaku, D‐MAX 2200 VPC) was applied to confirm their chemical compositions.

*Device Fabrication and Magnetoresistance Measurement*: In order to investigate the transport properties of these SmB_6_ NWs, four‐terminal devices were fabricated with standard e‐beam lithography and evaporation techniques. The electrodes consist of 10 nm Cr and 100 nm Au. The magnetotransport study of these SmB_6_ NWs was carried out in a 14T Quantum Design PPMS system, the base temperature of which is 2 K. Four‐probe resistivity was measured by the lock‐in technique. The amplitude and frequency of the excitation current is 100 nA and 375 Hz, respectively.

## Conflict of Interest

The authors declare no conflict of interest.

## Supporting information

Supplementary
