# Supplementary material for: Magnetoresistance Anomaly in Topological Kondo Insulator SmB6 Nanowires with Strong Surface Magnetism
Source: Adv Sci (Weinh). 2018 May 21;5(7):1700753. doi: 10.1002/advs.201700753 (PMC6051400; doi:10.1002/advs.201700753)
Supplement: Supplementary file 1 — Supplementary [file ADVS-5-1700753-s001.pdf]

Copyright WILEY-VCH Verlag GmbH & Co. KGaA, 69469 Weinheim, Germany, 2018.

ADVANCED SCIENCE
Open Access

Supporting Information

for *Adv. Sci.,* DOI: 10.1002/advs.201700753

Magnetoresistance Anomaly in Topological Kondo Insulator SmB_6 Nanowires with Strong Surface Magnetism

Xingshuai He, Haibo Gan, Zongzheng Du, Bicong Ye, Liang Zhou, Yuan Tian, Shaozhi Deng, Guoping Guo, Haizhou Lu, Fei Liu, and Hongtao He**

Copyright WILEY-VCH Verlag GmbH & Co. KGaA, 69469 Weinheim, Germany, 2016.

Supporting Information

Magnetoresistance Anomaly in Topological Kondo Insulator SmB_6 Nanowires with Strong Surface Magnetism

Xingshuai He, Haibo Gan, Zongzheng Du, Bicong Ye, Liang Zhou, Yuan Tian, Shaozhi Deng, Guoping Guo, Haizhou Lu, Fei Liu, and Hongtao He**

Robustness of the MR Anomaly under Thermal Cycling

We have also performed thermal cycling in one of our samples. The sample has a diameter of 52 nm. We first cooled the sample down to 2 K in a Quantum Design PPMS system and then measured the magnetotransport properties of it. The obtained magnetoresistance curve *R*(B) has been shown in Figure 5 in the main text. Besides this, we also measured the same sample in a dilution refrigerator system. Figure S1 shows the *R*(*B*) curve we obtained. It can be seen that the MR anomaly can still be observed, indicating the persistence of surface magnetism in SmB_6 nanowires under temperature cycling. It is also noted that the *R*(*B*) curve in Figure S1 shows differences to the one in Figure 5. Since the sample was exposed to air after it was taken out of the PPMS system and then put into the dilution refrigerator, the surface oxidation might reduce the effective diameter of the nanowire. As discussed in the main text, the MR anomaly depends on the diameter of the nanowire. Therefore, in comparison with the MR anomaly measured in the PPMS system, the MR anomaly measured in the dilution refrigerator gets enhanced.

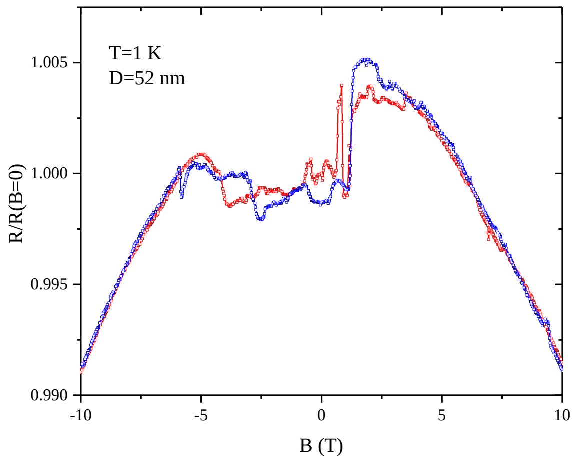

Figure S1. The *R*(*B*) curve of an SmB_6 nanowire with the diameter of 52 nm. The curve was measured at *T*=1 K in a dilution refrigerator system.
